# Effects of Glucoerb^®^ and Epavin^®^ on Cardiometabolic Profile in Adults with Overweight or Obesity: A Three-Month Real-World Study

**DOI:** 10.3390/jcm15197404

**Published:** 2026-09-23

**Authors:** Viviana Maggio, Francesca Vaccaro, Elena Muratore, Felicia Di Falco, Angelo Maria Patti, Mario Manunta, Vincenzo Geraci, Marco Chiaramonte, Carlotta Redavid, Anna Masucci, Marco Giammanco, Luisa Agnello, Marcello Ciaccio, Manfredi Rizzo

**Affiliations:** 1 School of Medicine, Department of Health Promotion, Maternal Childhood, Internal Medicine and Medical Specialties (PROMISE), University of Palermo, 90142 Palermo, Italy; viviana.maggio01@unipa.it (V.M.); francesca.vaccaro04@unipa.it (F.V.); elena.muratore@unipa.it (E.M.); felicia.difalco@unipa.it (F.D.F.); pattiangelomaria@gmail.com (A.M.P.); mariomanuntadiabetologo@gmail.com (M.M.); vincenzo.geraci@inwind.it (V.G.); marco.chiaramonte91@gmail.com (M.C.); carlotta.redavid3@gmail.com (C.R.); 2 Unit of Diabetes and Cardiometabolic Prevention, University Hospital of Palermo, 90127 Palermo, Italy; marco.giammanco@unipa.it; 3 Department of Biomedicine, Neuroscience and Advanced Diagnostic (BIND), University of Palermo, 90133 Palermo, Italy; anna.masucci@unipa.it (A.M.); luisa.agnello@unipa.it (L.A.); marcello.ciaccio@unipa.it (M.C.); 4 Institute of Clinical Biochemistry, Clinical Molecular Medicine, and Clinical Laboratory Medicine, University Hospital of Palermo, 90127 Palermo, Italy; 5 Department of Precision Medicine in Medical, Surgical and Critical Care (MePreCC), University of Palermo, 90133 Palermo, Italy

**Keywords:** obesity, insulin resistance, plant-based supplementation, cardiometabolic health, body composition, endothelial function, fatty liver index, lifestyle intervention

## Abstract

**Background/Objectives:** Overweight and obesity are major drivers of cardiometabolic dysfunction and increase the risk of type 2 diabetes (T2DM)and cardiovascular disease. Although lifestyle intervention remains the cornerstone of treatment, complementary strategies may provide additional benefits. The effects of Glucoerb^®^ and Epavin^®^, administered individually or in combination, on cardiometabolic health in adults with overweight or obesity remain poorly characterized. **Methods:** In this prospective real-world study, 150 adults received Glucoerb^®^ (*n* = 50), Epavin^®^ (*n* = 50), or their combination (*n* = 50), alongside individualized nutritional counselling based on a moderately carbohydrate-restricted Mediterranean diet and standardized physical activity recommendations. Anthropometric, body composition, biochemical, and vascular parameters were assessed at baseline and after 3 months. **Results:** All groups showed significant improvements in body weight, body mass index (BMI), waist circumference, and fat mass (FM) (all *p* < 0.001). Glucoerb^®^ supplementation was associated with a significant reduction in HOMA-IR (−21%), whereas Epavin^®^ supplementation improved the lipid profile (total cholesterol −13%, triglycerides−13%, LDL cholesterol −17%, HDL cholesterol +6%). Fatty Liver Index decreased across all groups (Glucoerb^®^ −14%, Epavin^®^ −19%, combination −17%). Combined supplementation was associated with reductions in body weight (−6%) and HOMA-IR (−32%), while endothelial function improved (+19%, *p* = 0.017). **Conclusions:** These findings support further investigation of complementary multi-target plant-based strategies for cardiometabolic risk management.

## 1. Background

Obesity is a chronic disease characterized by adipose tissue dysfunction, impaired metabolic homeostasis, and progressive cardiometabolic alterations [1,2]. Excess accumulation of visceral adipose tissue promotes adipocyte hypertrophy, dysregulated adipokine secretion, ectopic lipid deposition, and chronic low-grade inflammation, leading to systemic insulin resistance [3,4]. Insulin resistance represents a central pathophysiological link within this cardiometabolic continuum, impairing insulin action across multiple insulin-sensitive tissues and contributing to altered glucose homeostasis, atherogenic dyslipidemia, and progressive metabolic dysfunction [5,6]. These alterations contribute to systemic cardiometabolic complications, including Metabolic dysfunction-associated steatotic liver disease (MASLD), endothelial dysfunction, and cardiovascular disease [7,8].

Lifestyle interventions, including healthy dietary patterns, regular physical activity, and sustained weight management, remain the cornerstone of the prevention and treatment of obesity-related metabolic impairment [9]. Nevertheless, residual metabolic alterations frequently persist despite lifestyle intervention, particularly in individuals with overweight or obesity, highlighting the need for complementary therapeutic strategies [9]. The progression from obesity to overt type 2 diabetes (T2DM) is typically characterized by a prolonged phase of insulin resistance, during which metabolic dysfunction develops progressively. This stage represents a critical window for preventive interventions aimed at preserving metabolic health, delaying disease progression, and reducing long-term cardiometabolic risk [10,11].

In this context, nutraceuticals and naturally derived bioactive compounds have emerged as promising complementary strategies for the prevention and management of early cardiometabolic dysfunction [12,13]. Several bioactive compounds have been shown to exert pleiotropic effects on pathways involved in glucose homeostasis, insulin sensitivity, lipid metabolism, oxidative stress, inflammation, and hepatic metabolic function, suggesting a potential role in the management of early metabolic dysfunction [14,15,16,17]. This multi-target approach may be particularly relevant in individuals with insulin resistance, in whom early intervention may help improve metabolic impairment [16].

Recent evidence suggests that plant-derived bioactive compounds may modulate multiple pathways involved in obesity-related insulin resistance and cardiometabolic dysfunction. In particular, botanicals targeting glucose metabolism, including *Gymnema sylvestre*, *Momordica charantia*, and *Morus alba*, have demonstrated beneficial effects on glycemic control, insulin sensitivity, body weight, lipid profile, and endothelial function in individuals with impaired glucose metabolism, overweight, or obesity [18,19,20]. Complementary evidence indicates that plant-derived compounds with hepatometabolic activity, such as *Silybum marianum* and *Cynara scolymus*, improve hepatic steatosis, lipid metabolism, inflammatory biomarkers, endothelial function, and insulin resistance, supporting their potential role in the management of obesity-related cardiometabolic dysfunction [21,22,23,24,25]. 

Although several studies have demonstrated the metabolic benefits of individual plant-derived bioactive compounds, the clinical efficacy of combining bioactive compounds with complementary mechanisms of action warrants further investigation to establish whether this approach provides additional metabolic benefits in this high-risk population.

In this context, Glucoerb^®^ and Epavin^®^ are plant-based formulations intended to target complementary aspects of cardiometabolic health. Glucoerb^®^ is primarily formulated to support glucose metabolism and contains multiple plant-derived components, including *Momordica charantia*, *Lagerstroemia speciosa*, *Gymnema sylvestre*, *Cinnamomum zeylanicum*, *Silybum marianum*, and *Morus alba*, together with selected micronutrients [18,19,20]. Epavin^®^ contains multiple botanical extracts, including *Taraxacum officinale*, *Silybum marianum*, and *Cynara scolymus*, among others, that are potentially relevant to lipid and hepatic metabolism. Given their distinct but complementary compositions, their combined administration may provide a broader approach to cardiometabolic management. Notably, *Silybum marianum* is present in both formulations and may represent a common component contributing to their potential effects on lipid metabolism, insulin sensitivity, and hepatic metabolic pathways. 

However, the cardiometabolic effects of Glucoerb^®^ and Epavin^®^, administered individually and in combination within the same clinical setting, have not previously been investigated. To address this knowledge gap, the present study aimed to investigate the effects of a 3-month intervention with Glucoerb^®^, Epavin^®^, or their combination, administered alongside a personalized Mediterranean diet with moderate carbohydrate restriction and structured physical activity counseling, on body composition, anthropometric parameters, and cardiometabolic outcomes in adults with overweight or obesity.

## 2. Materials and Methods

### 2.1. Study Population

A total of 150 patients were consecutively recruited at the Diabetes and Cardiometabolic Prevention Unit of the University Hospital of Palermo between 1 December 2025 and 19 May 2026. Eligible participants were adults aged 18–70 years with a BMI ≥ 27 kg/m^2^. Exclusion criteria comprised type 1 or T2DM, severe hepatic or renal impairment, malignancy, severe infections, disabling systemic diseases, pregnancy, and breastfeeding. After enrolment, criteria for exclusion from the study included retracting of the informed consent, or failure to complete the 3-month follow-up. No patients were excluded from the study.

Written informed consent was obtained from all participants at enrolment. The procedures were conducted in accordance with the Declaration of Helsinki of 1975, as revised in 2013, and were approved by the Research Ethics Committee of the University Hospital of Palermo (approval date: 8 October 2025; protocol number: 23/2025). Following signed informed consent, a comprehensive medical history was obtained, and vital parameters, anthropometric measurements, vascular assessment, and laboratory parameters were systematically recorded for all participants. Patients were allocated into three intervention groups (*n* = 50 each) according to the nutraceutical supplementation prescribed: Glucoerb^®^, Epavin^®^, or the combination of both supplements. 

### 2.2. Study Formulations

Glucoerb^®^ and Epavin^®^ are commercially available plant-based formulations manufactured by Erbenobili Srl (Corato, Bari, Italy) and were provided free of charge to participants for the present study. Glucoerb^®^ and Epavin^®^ are classified and marketed in Italy as food supplements and are therefore subject to the notification procedure established by the Italian Ministry of Health for inclusion in the national Register of Food Supplements.

Glucoerb^®^ is a tablet formulation containing dry extracts of *Momordica charantia* L. fruit standardized to 3% bitter substances; *Lagerstroemia speciosa* (L.) Pers. leaves standardized to 1% corosolic acid; *Gymnema sylvestre* R. Br. leaves standardized to 25% gymnemic acid; *Cinnamomum zeylanicum* Blume bark; *Silybum marianum* (L.) Gaertn. fruit standardized to 80% silymarin; *Portulaca oleracea* L. aerial parts; *Morus alba* L. leaves standardized to 1% deoxynojirimycin; *Morinda citrifolia* L. freeze-dried juice; and *Vaccinium myrtillus* L. fruit dry extract standardized to 1% anthocyanidins. The formulation also contains zinc gluconate, cholecalciferol (vitamin D3), chromium picolinate, and D-biotin. Excipients include cellulose as a bulking agent and magnesium salts of fatty acids and silicon dioxide as anti-caking agents.

Epavin^®^ is a liquid formulation containing hydroalcoholic extracts of *Taraxacum officinale* (L.) Weber ex F.H. Wigg. root; *Silybum marianum* Gaertn. fruit; *Cynara scolymus* L. leaves; *Berberis vulgaris* L. root-derived bark; *Peumus boldus* Molina leaves; *Raphanus sativus* L. root; *Rheum palmatum* L. root; *Chrysanthellum americanum* (L.) *Vatke aerial* parts; *Desmodium adscendens* (Sw.) DC. leaves; and *Combretum micranthum* G. Don leaves. The formulation also contains essential oils of *Mentha × piperita* L. and *Rosmarinus officinalis* L.

According to the product information, Glucoerb^®^ was administered as four tablets daily before the main meals (two tablets before breakfast, one before lunch, and one before dinner), whereas Epavin^®^ was administered as 30 drops twice daily (2 mL/day), diluted in water or a warm beverage. Participants receiving the combination used both formulations according to the respective dosing regimens.

### 2.3. Anthropometric Assessment and Nutritional Evaluation

A comprehensive anthropometric and nutritional assessment was performed in all participants. Height and body weight were measured using calibrated equipment under standardized conditions. Body mass index (BMI) was calculated as weight (kg) divided by height squared (m^2^), and waist circumference was measured according to standardized procedures. Body composition was evaluated using bioelectrical impedance analysis (BIA; Akern BIA 101 BIVA PRO, Akern Srl, Pontassieve, Italy). Fat mass (FM), fat-free mass (FFM), skeletal muscle mass (SMM) and appendicular skeletal muscle mass (ASMM) were estimated from raw bioelectrical impedance parameters (resistance and reactance) [26].

Following the nutritional assessment, all participants received individualized nutritional counselling and were prescribed a personalized Mediterranean diet with moderate carbohydrate restriction, tailored to their nutritional requirements, metabolic profile, and clinical characteristics, according to the American Diabetes Association (ADA) Standards of Care in Diabetes [27]. Standardized physical activity counselling was also provided, encouraging participants to perform at least 150 min of moderate-intensity aerobic physical activity per week together with muscle-strengthening activities at least twice weekly, according to the World Health Organization recommendations [28].

### 2.4. Laboratory Analysis

Fasting venous blood samples were collected at the Laboratory Medicine Unit of the University Hospital of Palermo, and biochemical analyses were performed using standardized laboratory methods. The laboratory assessment included glycemic parameters, namely fasting plasma glucose, fasting insulin, glycated hemoglobin (HbA1c); lipid profile, including total cholesterol (TC), high-density lipoprotein cholesterol (HDL-C), low-density lipoprotein cholesterol (LDL-C), and triglycerides (TG); and inflammatory markers, including C-reactive protein (CRP). The Homeostatic Model Assessment for Insulin Resistance (HOMA-IR) was calculated using the validated formula based on fasting insulin and fasting glucose levels. The Fatty Liver Index (FLI) was calculated using the validated algorithm based on BMI, waist circumference, triglyceride levels, and gamma-glutamyl transferase [29]. All laboratory and clinical assessments were performed at baseline and repeated after the 3-month follow-up.

### 2.5. Endothelial Function Assessment

Endothelial function was evaluated by flow-mediated dilation (FMD) of the brachial artery, as previously described [30]. Briefly, a sphygmomanometer cuff was positioned 3–5 cm above the antecubital fossa and inflated to 25–30 mmHg above the participant’s systolic blood pressure for 5 min. Following cuff deflation, reactive hyperaemia was induced, and the brachial artery diameter was measured every 20 s over a 3-min period. Since maximal vasodilation is generally achieved between 60 and 90 s after cuff release, the highest recorded diameter was used for analysis. FMD was expressed as the percentage change in arterial diameter from baseline using the standard formula [31]. Endothelial function was assessed at baseline and reassessed after the 3-month follow-up.

### 2.6. Statistical Analysis

Continuous variables are presented as mean ± standard deviation (SD), whereas categorical variables are expressed as frequencies and percentages. Baseline differences among the three intervention groups were evaluated using one-way analysis of variance (ANOVA) for continuous variables and the chi-square test for categorical variables. Changes from baseline to the 3-month follow-up within each treatment group were assessed using the Wilcoxon signed-rank test. Percentage changes (% change) from baseline were also calculated for all study outcomes. A two-sided *p*-value < 0.05 was considered statistically significant. Statistical analyses were performed using Stata version 19 (StataCorp, College Station, TX, USA).

## 3. Results

### 3.1. Study Population Characteristics

A total of 150 participants were included in the study and classified into three groups according to the prescribed nutraceutical intervention: Group 1 (Glucoerb^®^, *n* = 50), Group 2 (Epavin^®^, *n* = 50), and Group 3 (combined Glucoerb^®^ and Epavin^®^, *n* = 50). The mean age was 54 ± 9 years in Group 1, 51 ± 13 years in Group 2, and 53 ± 12 years in Group 3. Women represented the majority of participants in all groups (70%, 86%, and 64% in Groups 1, 2, and 3, respectively). Overweight was more prevalent in Group 2 (60%) than in Group 1 (38%) and Group 3 (20%) (*p* < 0.001), whereas the highest prevalence of class III obesity was observed in Group 3 (18%, *p* = 0.024). No significant differences were found among the three groups in age, sex distribution, prevalence of dyslipidemia, hypertension, or previous cardiovascular events (Table 1).

No adverse events were reported in any of the three treatment groups during the three-month follow-up, and no participants discontinued supplementation due to tolerability issues.

### 3.2. Effects of the Study Interventions on Anthropometric and Body Composition Parameters

Table 2 summarizes the effects of the three study interventions on anthropometric and body composition parameters after 3 months of treatment. In Group 1, body weight significantly decreased from 85 ± 15 to 83 ± 14 kg (−3%, *p* < 0.001), accompanied by significant reductions in BMI (32 ± 5 vs. 31 ± 4 kg/m^2^, −3%, *p* < 0.001), waist circumference (101 ± 13 vs. 96 ± 12 cm, −4%, *p* < 0.001), and FM (33 ± 10 vs. 29 ± 10 kg, −13%, *p* < 0.001). Significant increases were also observed in FFM, MM, SMM, and ASMM. Similarly, Group 2 showed significant improvements in body weight (−2%), BMI (−3%), waist circumference (−5%), and FM (−12%) (*p* < 0.001 for all). In addition, FFM, MM, SMM, and ASMMsignificantly increased at the 3-month follow-up. The combined supplementation (Group 3) was associated with significant reductions in body weight (−6%), BMI (−3%), waist circumference (−6%), and FM (−13%) (*p* < 0.001 for all). FFM and SMMsignificantly increased, whereas MM and ASMM did not show statistically significant changes (Table 2).

### 3.3. Effects of Nutraceutical Interventions on Cardiometabolic Parameters and Endothelial Function

Table 3 summarizes the effects of the three nutraceutical interventions on cardiometabolic parameters after 3 months of treatment. In group 1, HOMA-IR significantly decreased by 21% (*p* = 0.013), whereas no significant changes were observed in fasting insulinemia or lipid profile. However, the FLI was significantly reduced by 14% (*p* < 0.001). Group 2 showed significant improvements in the lipid profile, including reductions in TC (−13%, *p* = 0.001), LDL-C (−17%, *p* < 0.001), and triglycerides (−13%, *p* = 0.021), together with a significant increase in HDL-C (+6%, *p* < 0.001). FLI also significantly decreased by 19% (*p* < 0.001), whereas no significant changes were observed in HOMA-IR or endothelial function. Group 3 showed a significant reduction in HOMA-IR (−32%, *p* = 0.006), TC (−4%, *p* < 0.001), LDL-C (−9%, *p* = 0.049), and FLI (−17%, *p* < 0.001). Moreover, endothelial function, assessed by FMD, significantly improved by 19% after treatment (*p* = 0.017), while no significant changes were observed in fasting insulinemia, triglycerides, HDL-C, or CRP (Table 3).

Representative changes observed in the three intervention groups are shown in Figure 1, Figure 2 and Figure 3.

## 4. Discussion

This three-month real-world study evaluated the effects of two plant-based formulations, Glucoerb^®^ and Epavin^®^, administered individually or in combination, in individuals with overweight or obesity undergoing a structured lifestyle intervention. Although several plant-derived bioactive compounds have been individually investigated for their potential metabolic effects, the available clinical evidence has largely focused on single components or isolated metabolic outcomes. Evidence regarding multi-component plant-based formulations combining different bioactive compounds within a single supplementation strategy remains comparatively limited, particularly in the context of a comprehensive cardiometabolic assessment. Therefore, the present study addresses this gap by evaluating Glucoerb^®^ and Epavin^®^, administered individually and in combination, within an integrated assessment of the cardiometabolic profile of adults with overweight or obesity. This approach provides a broader perspective on the potential role of complex plant-based supplementation strategies in cardiometabolic health.

The lifestyle intervention implemented across all treatment groups provided a consistent clinical background for the evaluation of supplementation-related changes. Overall, the findings support an integrated approach in which lifestyle modification represents the foundation of cardiometabolic management, while plant-based supplementation may provide complementary metabolic benefits. This aspect is consistent with growing evidence supporting plant-derived bioactive compounds as components of comprehensive metabolic management rather than isolated therapeutic agents.

Significant improvements were observed across all three intervention groups (with Glucoerb^®^, Epavin^®^ or Combination), with reductions in body weight (−3%, −2%, and −6%, respectively), waist circumference (−4%, −5%, and −6%), and FM (−13%, −12%, and −13%) (all *p* < 0.001), reflecting consistent changes across multiple anthropometric and body composition parameters. Although the absolute weight loss was modest, its clinical relevance should not be underestimated. Previous studies have consistently demonstrated that a reduction of as little as 3–5% of initial body weight is associated with meaningful improvements in insulin sensitivity, glucose metabolism, blood pressure, and cardiovascular risk [32]. An additional finding of particular interest was the preservation of FFM (from +1% to +4% among the different study groups) and SMM (from +2% to +8%) despite the reduction in body weight. This suggests that the intervention preferentially promoted adipose tissue loss while maintaining lean tissue, a body composition profile associated with improved long-term metabolic health. Preservation of MM is especially important in individuals with obesity, as skeletal muscle plays a central role in glucose disposal, insulin sensitivity, and resting energy expenditure [33,34,35]. Collectively, these findings indicate that combining lifestyle intervention with nutraceutical supplementation may improve body composition beyond simple weight reduction, promoting metabolically favorable changes that extend beyond reductions in total body mass.

Among all metabolic outcomes evaluated, improvements in insulin sensitivity emerged as one of the most clinically relevant findings. HOMA-IR decreased significantly following Glucoerb^®^ supplementation (−21%, *p* = 0.013) and combined treatment (−32%, *p* = 0.006). Since insulin resistance represents the central pathophysiological abnormality underlying metabolic syndrome and a major predictor of T2DM progression, these results carry important clinical implications. The observed insulin-sensitizing effects of Glucoerb^®^ may be related to its bioactive compounds targeting glucose homeostasis. *Momordica charantia* and *Gymnema sylvestre*, two key components of Glucoerb^®^, have demonstrated beneficial effects on glycaemic control and insulin sensitivity in individuals with impaired glucose metabolism [18,19,36]. *Morus alba* further supports glucose metabolism through alpha-glucosidase inhibition, while *Silybum marianum* contributes hepatoprotective and insulin-sensitizing effects [21,37]. In parallel, carbohydrate restriction and regular physical activity independently improve insulin sensitivity by decreasing hepatic glucose output, reducing circulating free fatty acids, and enhancing skeletal muscle glucose utilization [38]. The reduction in HOMA-IR observed with combined supplementation occurred alongside the metabolic adaptations associated with lifestyle modification. Interestingly, although Epavin^®^ alone was not associated with a significant change in HOMA-IR, a significant reduction was observed in the combination group. This observation may reflect the involvement of multiple pathways related to glucose metabolism, lipid regulation, oxidative stress, and vascular homeostasis. Improvements in lipid metabolism and systemic inflammation may reduce lipotoxicity and improve insulin signaling, potentially contributing to the metabolic effects of Glucoerb^®^. Such complementary mechanisms are consistent with the current understanding that insulin resistance is a multifactorial process requiring interventions capable of targeting multiple biological pathways simultaneously [39,40].

The observed changes in lipid parameters may reflect distinct but potentially complementary effects of the two plant-based formulations. Epavin^®^ supplementation was associated with significant improvements in the lipid profile, with significant reductions in TC (−13%, *p* = 0.001), LDL-C (−17%, *p* < 0.001), and triglycerides (−13%, *p* = 0.021), together with a significant increase in HDL-C (+6%, *p* < 0.001). These findings are consistent with the well-established lipid-modulating properties of plant-derived polyphenols and the hepatoprotective compounds contained in Epavin^®^. *Silybum marianum* and *Cynara scolymus* have been shown to regulate cholesterol homeostasis, enhance reverse cholesterol transport, reduce hepatic lipogenesis, and improve lipid metabolism [21,22,24,41]. Additionally, the herbal extract combination in Epavin^®^, including *Taraxacum officinale*, *Desmodium adscendens*, and *Combretum micranthum,* supports hepatic function and lipid handling through multiple metabolic pathways [42]. Low-carbohydrate dietary patterns are known to reduce hepatic very-low-density lipoprotein production and improve triglyceride metabolism, while exercise enhances lipoprotein lipase activity and HDL synthesis [43,44]. The changes observed with Epavin^®^ occurred alongside these lifestyle-related metabolic adaptations and were associated with an overall improvement in the lipid profile. The combined supplementation group also showed significant reductions in TC (−4%, *p* < 0.001) and LDL-C(−9%, *p* = 0.049). This may reflect the broader metabolic activity of the combined intervention, which simultaneously targeted glucose metabolism, insulin sensitivity, hepatic steatosis, and vascular function. In this context, the combination therapy appears to distribute its metabolic effects across multiple physiological systems instead of maximizing a single outcome. Such a multidimensional response may be particularly advantageous in individuals presenting with the complex metabolic abnormalities characteristic of obesity and metabolic syndrome.

The FattFLI), a surrogate marker of hepatic steatosis, improved significantly in all treatment groups (−14%, −19% and −17%, respectively, *p* < 0.001 for all), reflecting meaningful improvements in hepatic metabolic health. The improvement in hepatic steatosis is likely multifactorial. Weight loss, even of modest magnitude, has been consistently associated with reductions in hepatic fat accumulation. Likewise, improved insulin sensitivity decreases hepatic de novo lipogenesis while promoting fatty acid oxidation. Beyond lifestyle modification, nutraceutical supplementation may have provided additional hepatoprotective effects through complementary mechanisms. Both Glucoerb^®^ and Epavin^®^ contain *Silybum marianum*, which has well-documented hepatoprotective properties and improves hepatic steatosis through antioxidant mechanisms and enhanced lipid metabolism [21,22,23,24]. The polyphenolic compounds present in both formulations possess antioxidant and anti-inflammatory properties capable of attenuating oxidative stress and inflammatory pathways involved in the progression of fatty liver disease [45]. Since hepatic steatosis is increasingly recognized not only as a consequence but also as a driver of systemic metabolic dysfunction, improvements in liver health significantly contribute to the broader cardiometabolic benefits observed throughout the study.

Regarding the inflammatory profile, changes in CRP levels were also evaluated. CRP decreased numerically in all three intervention groups (Glucoerb^®^: −13%, *p* = 0.979; Epavin^®^: −9%, *p* = 0.718; combination: −38%, *p* = 0.292), although none of these changes reached statistical significance. The potential anti-inflammatory effects of Glucoerb^®^ may be related to its polyphenolic content and other bioactive compounds derived from *Momordica charantia* and *Gymnema sylvestre*, which may modulate inflammatory pathways and reduce oxidative stress [46]. Epavin^®^, through its rich polyphenolic extract and hepatoprotective components, including *Silybum marianum, Cynara scolymus*, and supporting herbal agents, contributes to reductions in hepatic and systemic inflammation by improving hepatic detoxification capacity and reducing endotoxemia-related inflammatory signals [21,24,41]. The combined formulation therefore likely provides redundant anti-inflammatory mechanisms targeting both metabolic inflammation and hepatic inflammatory pathways. Although the CRP reductions did not achieve statistical significance, the magnitude of change with combination therapy (−38%) suggests a potential clinical benefit that may become more apparent with extended follow-up or larger sample sizes, particularly in populations with elevated baseline inflammatory markers.

One of the most noteworthy findings of the present study was the significant improvement in FMDobserved in the combination group (+19%, *p* = 0.017), whereas no significant changes were observed in the other two groups. As FMD represents a validated surrogate marker of endothelial function and an early indicator of vascular health, this finding suggests a potential beneficial effect of combined supplementation on endothelial function, extending beyond the observed metabolic changes [41]. Several complementary mechanisms may explain this finding. Improved insulin sensitivity enhances endothelial nitric oxide synthase activity and nitric oxide bioavailability, thereby promoting vasodilation [47]. At the same time, polyphenolic compounds present in both Glucoerb^®^ and Epavin^®^, particularly the anthocyanidins and other antioxidant compounds, have consistently demonstrated the capacity to reduce oxidative stress, improve endothelial signalling, and preserve vascular function [48]. Lifestyle modification likely contributed additional benefits through reductions in postprandial glycaemic excursions, improved metabolic flexibility, and exercise-induced increases in endothelial shear stress, all of which stimulate nitric oxide production. The vascular changes observed in the combination group support the close interconnection between metabolic and vascular health and suggest that multiple mechanisms may be involved in the observed improvement in endothelial function. Taken together, the present findings support the concept that effective management of early cardiometabolic dysfunction requires interventions targeting multiple interconnected metabolic pathways. Rather than replacing lifestyle modification, nutraceutical supplementation should be considered an adjunctive strategy capable of enhancing the metabolic adaptations induced by dietary intervention and regular physical activity. Whether combined supplementation provides additional benefits over the individual formulations requires evaluation in appropriately designed comparative trials.

Regarding tolerability, no adverse events were reported in any of the three treatment groups, and no participants discontinued supplementation due to tolerability issues. These findings suggest that both plant-based formulations, administered individually or in combination, were well tolerated over the three-month study period. From a broader clinical perspective, these interventions may be particularly relevant for adults with overweight or obesity presenting with insulin resistance, dyslipidemia, central adiposity, and an elevatedFLI. In this syndromic profile, the improvements observed after three months suggest a potential role for nutraceutical supplementation as an adjunct to lifestyle intervention in the short-term management of cardiometabolic risk. However, given the limited duration of the present study, whether these benefits are maintained over the medium and long term remains to be established. Longer-term controlled studies are therefore needed to determine the persistence of these metabolic effects and their potential impact on the progression of cardiometabolic disease. These plant-based formulations could potentially be integrated with pharmacological therapy, when clinically indicated, as part of a comprehensive approach to cardiometabolic risk management; however, this potential adjunctive role requires evaluation in specifically designed prospective studies.

Nevertheless, several limitations should also be considered. Baseline heterogeneity was observed across the three treatment groups, particularly in the distribution of overweight and obesity classes, reflecting the real-world nature of the study and the clinical characteristics of participants receiving the different study interventions. The three-month duration allowed the assessment of short-term metabolic changes but does not permit conclusions regarding the long-term sustainability of the observed effects. Longer follow-up studies are therefore required to determine whether these early metabolic improvements translate into durable clinical benefits. Furthermore, individual quantitative data on dietary intake and physical activity during follow-up were not available, precluding covariate-adjusted analyses for these lifestyle factors. Therefore, potential differences in dietary intake and adherence to physical activity recommendations cannot be excluded. Finally, the study population consisted primarily of individuals with overweight or class I obesity and relatively early metabolic dysfunction. While this may limit generalizability to patients with more advanced obesity, it also represents a major strength from a preventive perspective.

## 5. Conclusions

In conclusion, this real-world study suggests that structured lifestyle intervention combined with plant-based supplementation was associated with significant improvements in multiple domains of cardiometabolic health. The individual or combined administration of Glucoerb^®^ and Epavin^®^ was associated with improvements across several clinically relevant outcomes. Although larger randomized controlled trials with longer follow-up are needed to confirm these observations, the current findings support further investigation of plant-based supplementation as a potential adjunct to comprehensive lifestyle strategies for the early management of overweight or obesity and associated cardiometabolic alterations. These formulations could potentially be integrated with pharmacological therapy, when clinically indicated, as part of a comprehensive approach to cardiometabolic risk management. Further studies are warranted to evaluate this potential adjunctive role and to determine whether the observed cardiometabolic benefits are sustained over the medium and long term. 

## Figures and Tables

**Figure 1:**
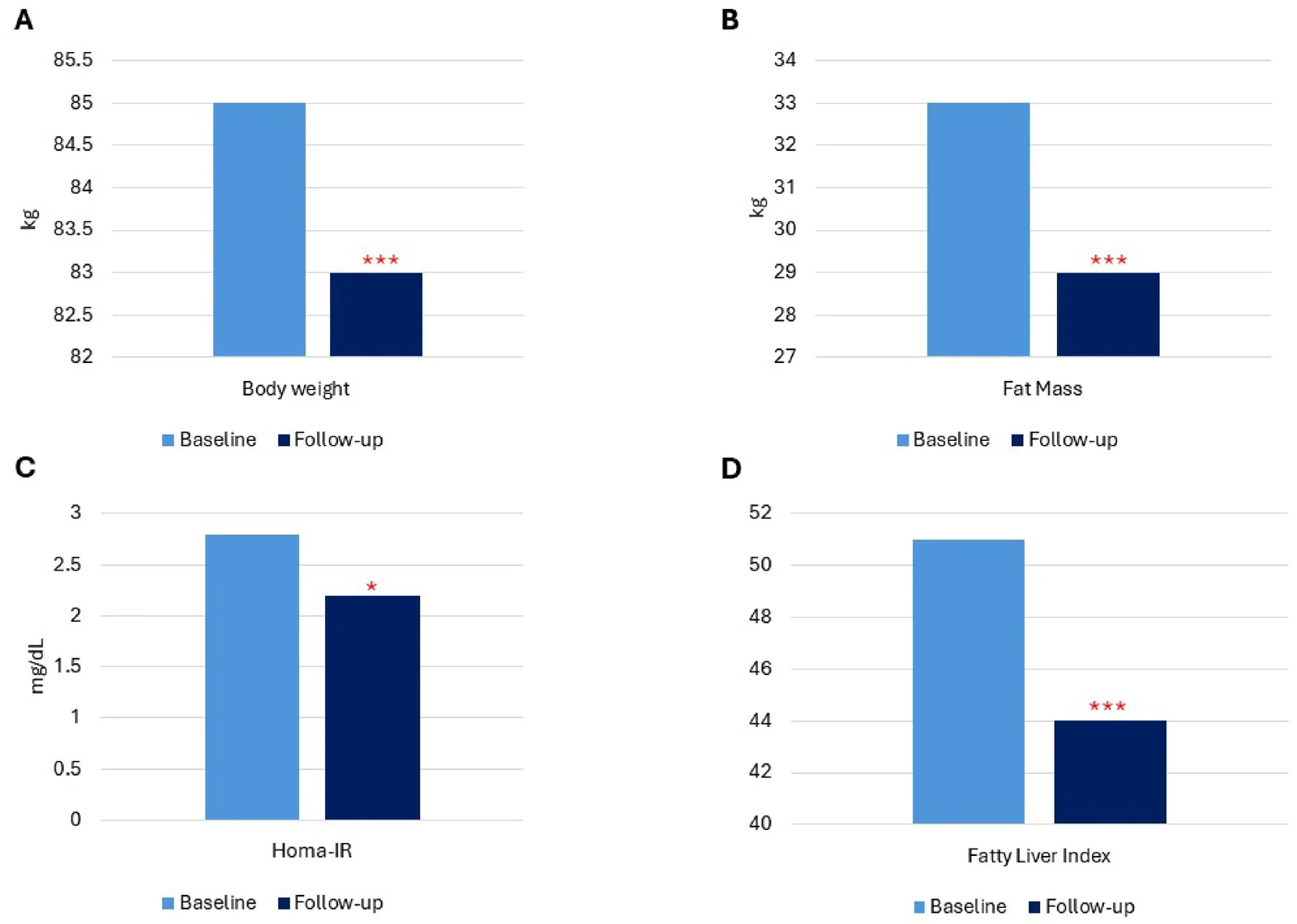
Changes in anthropometric and metabolic parameters following 3 months of Glucoerb^®^ supplementation. (**A**) Body weight; (**B**) fat mass; (**C**) HOMA-IR; (**D**) Fatty Liver Index (FLI). * *p* < 0.05, *** *p* < 0.001.

**Figure 2:**
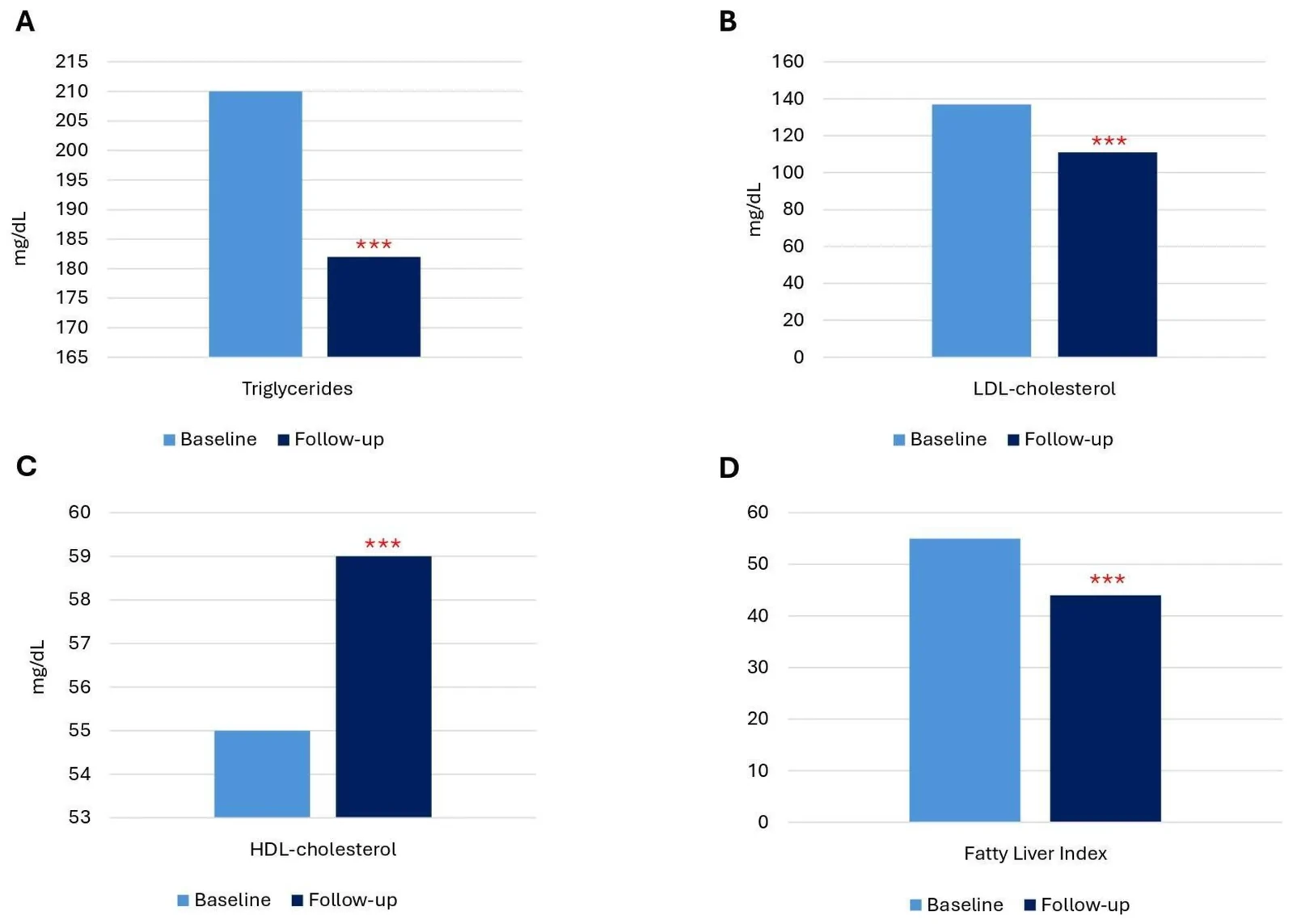
Changes in lipid profile parameters following 3 months of Epavin^®^ supplementation. (**A**) Triglycerides (mg/dL); (**B**) LDL-cholesterol (mg/dL); (**C**) HDL-cholesterol (mg/dL); (**D**) Fatty Liver Index., *** *p* < 0.001.

**Figure 3:**
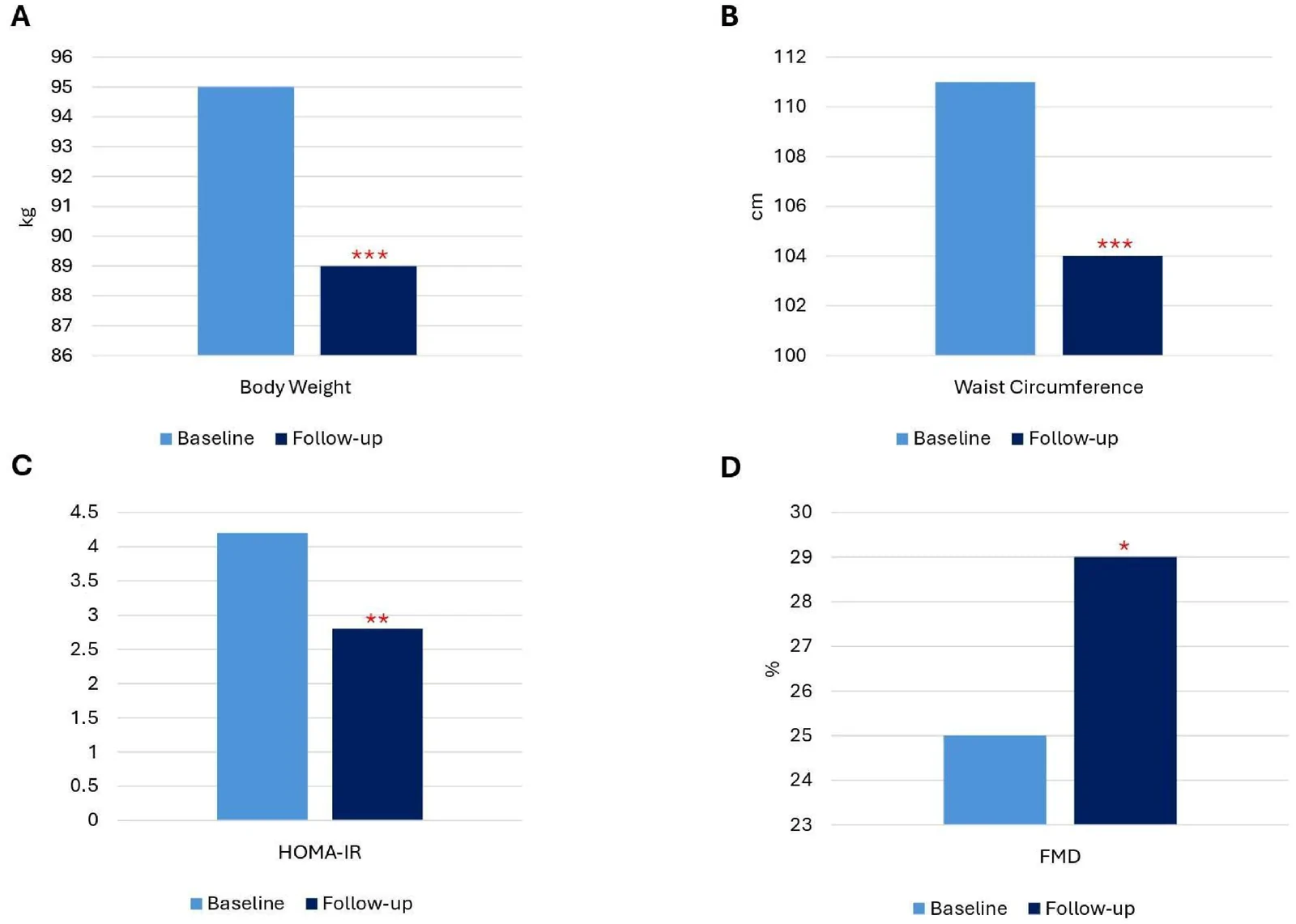
Changes in anthropometric, metabolic, and inflammatory parameters following 3 months of combined Glucoerb^®^ and Epavin^®^ supplementation. (**A**) Body weight (kg); (**B**) waist circumference (cm); (**C**) HOMA-IR; (**D**) C-reactive protein (CRP) (mg/L). * *p* < 0.05, ** *p* < 0.01, *** *p* < 0.001.

**Table 1:** Baseline demographic, anthropometric, and clinical characteristics of the study participants (*n* = 150).

Variable (Mean ± SD or *n*, %)	Group 1 with Glucoerb^®^ (*n* = 50)	Group 2 with Epavin^®^ (*n* = 50)	Group 3 with Glucoerb^®^ + Epavin^®^ (*n* = 50)	*p*-Value
Age (years)	54 ± 9	51 ± 13	53 ± 12	0.112
Women, *n* (%)	35 (70)	43 (86)	32 (64)	0.101
Overweight, *n* (%)	19 (38)	30 (60)	10 (20)	<0.001
Obesity class I, *n* (%)	23 (46)	13 (26)	17 (34)	0.459
Obesity class II, *n* (%)	6 (12)	6 (12)	14 (28)	0.139
Obesity class III, *n* (%)	2 (4)	1 (2)	9 (18)	0.024
Dyslipidemia, *n* (%)	20 (40)	40 (80)	32 (64)	0.060

Data are presented as mean ± standard deviation (SD) for continuous variables and as number (percentage) for categorical variables. Between-group comparisons were performed using one-way analysis of variance (ANOVA) for continuous variables and the chi-square test for categorical variables. A two-sided *p*-value < 0.05 was considered statistically significant.

**Table 2:** Effects of study interventions on anthropometric and body composition parameters in subjects with overweight or obesity. Data are expressed as mean ± SD.

	Group 1 with Glucoerb^®^ (*n* = 50)		% Change and *p*-Value	Group 2 with Epavin^®^ (*n* = 50)		% Change and *p*-Value	Group 3 with Glucoerb^®^ + Epavin^®^ (*n* = 50)		% Change and *p*-Value
	Baseline	Follow-Up		Baseline	Follow-Up		Baseline	Follow-Up	
Body Weight (kg)	85 ± 15	83 ± 14	−3 (<0.001)	83 ± 15	81 ± 15	−2 (<0.001)	95 ± 20	89 ± 17	−6 (<0.001)
Body Mass Index (kg/m^2^)	32 ± 5	31 ± 4	−3 (<0.001)	31 ± 4	30 ± 4	−3 (<0.001)	35 ± 6	34 ± 6	−3 (<0.001)
Waist Circumference (cm)	101 ± 13	96 ± 12	−4 (<0.001)	100 ± 12	96 ± 12	−5 (<0.001)	111 ± 14	104 ± 13	−6 (<0.001)
Fat Mass (kg)	33 ± 10	29 ± 10	−13 (<0.001)	28 ± 9	25 ± 9	−12 (<0.001)	38 ± 13	33 ± 11	−13 (<0.001)
Fat-free Mass (kg)	52 ± 8	54 ± 10	+2 (<0.001)	55 ± 11	56 ± 11	+1 (<0.001)	53 ± 12	55 ± 10	+4 (0.049)
Muscle Mass (kg)	33 ± 6	34 ± 7	+2 (0.010)	36 ± 8	36 ± 8	+2 (0.030)	36 ± 9	37 ± 9	+3 (0.179)
Skeletal Muscle Mass (kg)	22 ± 5	24 ± 6	+6 (<0.001)	24 ± 7	26 ± 7	+8 (0.010)	25 ± 7	26 ± 7	+4 (<0.001)
Appendicular Skeletal Muscle Mass (kg)	20 ± 4	21 ± 4	+2 (0.007)	21 ± 5	22 ± 5	+5 (0.030)	22 ± 5	23 ± 5	+4 (0.517)

Data are presented as mean ± standard deviation (SD). Within-group comparisons between baseline and 3-month follow-up were performed using the Wilcoxon signed-rank test. A two-sided *p*-value < 0.05 was considered statistically significant.

**Table 3:** Effects of study interventions on cardiometabolic parameters in subjects with overweight or obesity.

	Group 1 with Glucoerb^®^ (*n* = 50)		% Change and *p*-Value	Group 2 with Epavin^®^ (*n* = 50)		% Change and *p*-Value	Group 3 with Glucoerb^®^ + Epavin^®^ (*n* = 50)		% Change and *p*-Value
	Baseline	Follow-Up		Baseline	Follow-Up		Baseline	Follow-Up	
Insulin (mIU/L)	12 ± 6	11 ± 5	−8 (0.248)	13 ± 9	11 ± 5	−12 (0.304)	17 ± 10	14 ± 7	−18 (0.122)
HOMA-IR	2.8 ± 1.5	2.2 ± 1.5	−21	3.0 ± 2.5	2.5 ± 1.4	−17	4.2 ± 2.7	2.8 ± 2.0	−32
			(0.013)			(0.126)			(0.006)
TC (mg/dL)	185 ± 39	179 ± 32	−3	210 ± 51	182 ± 44	−13	183 ± 45	176 ± 33	−4
			(0.504)			(0.001)			(<0.001)
TG (mg/dL)	93 ± 38	86 ± 43	−7	112 ± 59	98 ± 54	−13	113 ± 47	108 ± 51	−4.2
			(0.369)			(0.021)			(0.329)
LDL (mg/dL)	114 ± 38	110 ± 29	−3 (0.658)	137 ± 45	113 ± 40	−17 (<0.001)	124 ± 39	113 ± 30	−9 (0.049)
HDL (mg/dL)	58 ± 12	59 ± 12	+2 (0.381)	55 ± 13	59 ± 13	+6 (<0.001)	51 ± 16	53 ± 15	+4 (0.607)
C-reactive protein (mg/L)	3.9 ± 6.6	3.4 ± 4.2	−13 (0.979)	2.1 ± 2.1	1.9 ± 1.8	−9 (0.718)	4.5 ± 5.2	2.8 ± 3.4	−38 (0.292)
Fatty Liver	51 ± 29	44 ± 26	−14 (<0.001)	55 ± 27	44 ± 28	−19 (<0.001)	74 ± 24	61 ± 28	−17
Index									(<0.001)
Flow-mediated dilation (%)	26 ± 21	27 ± 19	+1 (0.950)	25 ± 6	29 ± 15	+18 (0.285)	25 ± 11	29 ± 17	+19 (0.017)

Data are presented as mean ± standard deviation (SD). Within-group comparisons between baseline and 3-month follow-up were performed using the Wilcoxon signed-rank test. A two-sided *p*-value < 0.05 was considered statistically significant.

## Data Availability

The data presented in this study are available from the corresponding author upon reasonable request. The data are not publicly available because they contain information that could compromise participant privacy.
